# The T box regulatory element controlling expression of the class I lysyl-tRNA synthetase of *Bacillus cereus *strain 14579 is functional and can be partially induced by reduced charging of asparaginyl-tRNA^Asn^

**DOI:** 10.1186/1471-2180-10-196

**Published:** 2010-07-22

**Authors:** Niall Foy, Brian Jester, Gavin C Conant, Kevin M Devine

**Affiliations:** 1 Smurfit Institute of Genetics, Trinity College Dublin, Dublin 2. Ireland; 2 Institut de Biologie Moléculaire et Cellulaire, 15 Rue René Descartes, 67 084 Strasbourg, France; 3 Division of Animal Sciences and Informatics Institute, University of Missouri, Columbia, MO 65211. USA

## Abstract

**Background:**

Lysyl-tRNA synthetase (LysRS) is unique within the aminoacyl-tRNA synthetase family in that both class I (LysRS1) and class II (LysRS2) enzymes exist. LysRS1 enzymes are found in *Archaebacteria *and some eubacteria while all other organisms have LysRS2 enzymes. All sequenced strains of *Bacillus cereus *(except AH820) and *Bacillus thuringiensis *however encode both a class I and a class II LysRS. The *lysK *gene (encoding LysRS1) of *B. cereus *strain 14579 has an associated T box element, the first reported instance of potential T box control of LysRS expression.

**Results:**

A global study of 891 completely sequenced bacterial genomes identified T box elements associated with control of LysRS expression in only four bacterial species: *B. cereus, B. thuringiensis, Symbiobacterium thermophilum *and *Clostridium beijerinckii*. Here we investigate the T box element found in the regulatory region of the *lysK *gene in *B. cereus *strain 14579. We show that this T box element is functional, responding in a canonical manner to an increased level of uncharged tRNA^Lys ^but, unusually, also responding to an increased level of uncharged tRNA^Asn^. We also show that *B. subtilis *strains with T box regulated expression of the endogenous *lysS *or the heterologous *lysK *genes are viable.

**Conclusions:**

The T box element controlling *lysK *(encoding LysRS1) expression in *B. cereus *strain 14579 is functional, but unusually responds to depletion of charged tRNA^Lys ^and tRNA^Asn^. This may have the advantage of making LysRS1 expression responsive to a wider range of nutritional stresses. The viability of *B. subtilis *strains with a single LysRS1 or LysRS2, whose expression is controlled by this T box element, makes the rarity of the occurrence of such control of LysRS expression puzzling.

## Background

The aminoacyl tRNA synthetase (AARS) family of enzymes function to attach amino acids to their cognate tRNAs [1-3]. Each enzyme specifically charges a tRNA with its cognate amino acid in an energy requiring reaction that is executed with very high fidelity. However, despite all AARSs carrying out essentially the same reaction, the AARS family is subdivided into class I and class II enzymes that are structurally distinct and unrelated phylogenetically [for reviews see [3,4]]. This division of AARS into class I and class II enzymes is universal with each AARS being a member of one or other enzyme class in all living organisms. The lysyl-tRNA synthetase (LysRS) is an exception in that both class I (LysRS1) and class II (LysRS2) variants exist [5,6]. LysRS1 enzymes are found in *Archaebacteria *and in some eubacteria (*eg*. *Borrelia *and *Treponema *species) while LysRS2 enzymes are found in most eubacteria and all eukaryotes. Interestingly some bacteria have both class I LysRS1 and class II LysRS2 enzymes. For example, in *Methanosarcina barkeri *the class I and class II LysRS enzymes function as a complex to charge tRNA^Pyl ^with the rare pyrolysine amino acid while in *B. cereus *strain 14579 both enzymes can function together to aminoacylate a small tRNA-like molecule (tRNA^Other^) that functions to control expression TrpRS1 [7-9].

Sustaining charged tRNAs at levels adequate for the protein synthetic needs of growth under each environmental and nutritional condition is crucial for cell survival. Achieving this mandates that expression of each AARS be responsive to the cellular level of their charged cognate tRNAs. Therefore the mechanisms controlling AARS expression must be able to distinguish their cognate tRNA from other tRNA species and be able to measure the extent to which the pool of cognate tRNA is charged. Expression of the majority of AARSs in *Bacillus subtilis *is regulated by the T box antitermination mechanism [10]. This mechanism was first discovered in studies on the regulation of threonyl- and tyrosyl-tRNA synthetase expression in *B. subtilis *[11-13], for a review see 14. The T box elements are widely distributed, being present in *Firmicutes, δ-proteobacteria, Chloroflexi, Deinococcales/Thermales *and *Actinobacteria*, and control expression of genes involved in cellular activities other than tRNA charging such as amino acid biosynthesis, amino acid transport and regulation of amino acid metabolism [15-17].

The T-box regulatory element is usually a 200-300 nucleotide untranslated RNA leader sequence containing a conserved T box sequence, stem-loop structures and a conditional Rho-independent terminator located upstream of the start codon [11-13]. Two specific interactions between tRNAs and T box leader sequences enable recognition of cognate tRNA species and distinction between charged and uncharged pools of tRNA. The NCCA sequence in the acceptor stem of a nonacylated-tRNA interacts with the UGGN sequence within the T box sequence (N varies according to the identity of the discriminator base of each tRNA) [13,14,18,19]. This interaction cannot occur when a tRNA is aminoacylated, thereby distinguishing between charged and uncharged tRNAs. Specificity for cognate tRNAs is achieved by the presence of a specifier codon within a bulge in stem I of the leader sequence that interacts with the anticodon sequence of each tRNA. (*eg. *See Additional file 1, Figure S5). Thus for T box control of AARS expression, a high level of an uncharged tRNA (necessitating increased AARS production) causes interaction between that tRNA and its cognate T box element that stabilizes the anti-termination structure of the leader sequence allowing transcription of the AARS gene to proceed. A high level of aminoacylated-tRNAs in contrast cannot interact with the leader sequence allowing formation of the Rho-independent terminator and preventing continued transcription of the gene.

While most eubacteria encode either a class I or a class II LysRS, all sequenced strains of *B. cereus *(except strain AH820) and *B. thuringiensis *encode a copy of both enzyme types [8,16,17]. In *Bacillus cereus *strain 14579, the LysRS2-encoding *lysS *gene is positioned at the end of an operon encoding genes involved in folate metabolism, its normal position in most *Bacilli *while the *lysK *gene encoding the class I-type LysRS1 is located elsewhere on the chromosome. Shaul *et al. *(2006) show that this LysRS1 is closely related to the class I LysRS1 of *Pyrococcus*, suggesting that it has been acquired by *B. cereus *by horizontal transfer [20]. The function of LysRS1 in *B. cereus *is not clear but it is expressed predominantly in stationary phase and can aminoacylate a novel tRNA species (tRNA^Other^) in concert with the class II LysRS enzyme [8]. Thus it may play a role in surviving nutritional downshift in *B. cereus*.

Ataide and colleagues reported the presence of a putative T box regulatory element upstream of the *lysK *coding sequence in *B. cereus *strain 14579 [8]. This was the first reported instance of putative control of LysRS expression by a T box mechanism. Here we investigate control of LysRS expression by a T box mechanism, confirming that it occurs only very rarely in bacteria. We show that the T box element of the *lysK *gene of *B. cereus *strain 14579 is functional and responds to an increased level of uncharged tRNA^Lys ^in a canonical manner. Interestingly, this T box element shows some promiscuity in its specificity by responding to a reduced cellular level of asparaginyl-tRNA^Asn^. We also show that strains of *B. subtilis*, in which expression of the endogenous LysRS2 or the heterologous LysRS1 is controlled by this T box element, are viable.

## Results

### Regulation of lysyl tRNA synthetase expression by a T-box antitermination mechanism occurs rarely

A search of the upstream region of AARS-encoding genes in 891 completely sequenced bacterial genomes identified 976 T box elements. Significant variation in the frequency with which individual AARS are regulated by a T box mechanism was observed in this cohort, consistent with previous reports [16,17]. Control of LysRS expression by T box elements occurs very rarely, being documented in only 4 bacterial species: all sequenced *B. cereus *strains (except AH820); in *B. thuringiensis *strains Konkukian and Al Hakam; in *Clostridium beijerinckii *and in *Symbiobacterium thermophilum *[8,16,17]. These cases display several interesting features (Table 1): (i) all bacterial species with T-box regulated LysRS expression have a second LysRS that is not T-box regulated; (ii) the phylogenetically related *B. cereus *and *B. thuringiensis *species each have a class II LysRS2 and a T-box regulated class I LysRS1 - these T box regulatory elements show very high sequence conservation (~92% identity, Additional file 1, Figures S1, S5); (iii) conversely in *S. thermophilum*, the class II LysRS2 (STH525) is regulated by a T box element with little similarity to that found in the *Bacillus *species (Additional file 1, Figures S3, S7) while the class I LysRS1 (STH208) is not T box regulated and (iv) *C. beijerincki *has two classII LysRS (Cbei_3591 and Cbei_0105), one of which (Cbei_3591) is regulated by a T box element that displays clear sequence similarity (~50% identity) to the T box found in the *Bacillus *species (see Additional file 1, Figures S2, S6), but little similarity to the T box element of *S. thermophilum *(Additional file 1, Figure S4). Thus T box regulated LysRS expression is very rare and is invariably accompanied by a second non-T-box regulated (either class I or class II) LysRS. Two separate T box elements were identified - one controlling expression of a class II LysRS2 in *S. thermophilum *and the second controlling expression of a class I LysRS1 in *B. cereus *and *B. thuringiensis *but a class II LysRS2 in *C. beijerinckii*

**Table 1:** Occurrence of T box regulated lysyl-tRNA synthetase genes

Bacterium	Gene	Gene identifier	Class	T box regulated
*B. cereus *strains§	*lysS*		II	No
	*lysK*		I	Yes
*B. thuringiensis *Konkukian	*lysS*	BT9727_0072	II	No
	*lysK*	BT9727_2375	I	Yes
*B. thuringiensis *Al Hakam	*lysS*	BALH_0075	II	No
	*lysK*	BALH_2333	I	Yes
*Clostridium beijerinckii*	*lysS1*	Cbei_0105	II	No
	*lysS2*	Cbei_3591	II	Yes
*Symbiobacterium thermophilum*	*lysS*	STH525	II	Yes
	*lysK*	STH208	I	No

### The T-box element controlling expression of *lysK *in *B. cereus *strain 14579 is functional

**Figure 1:**
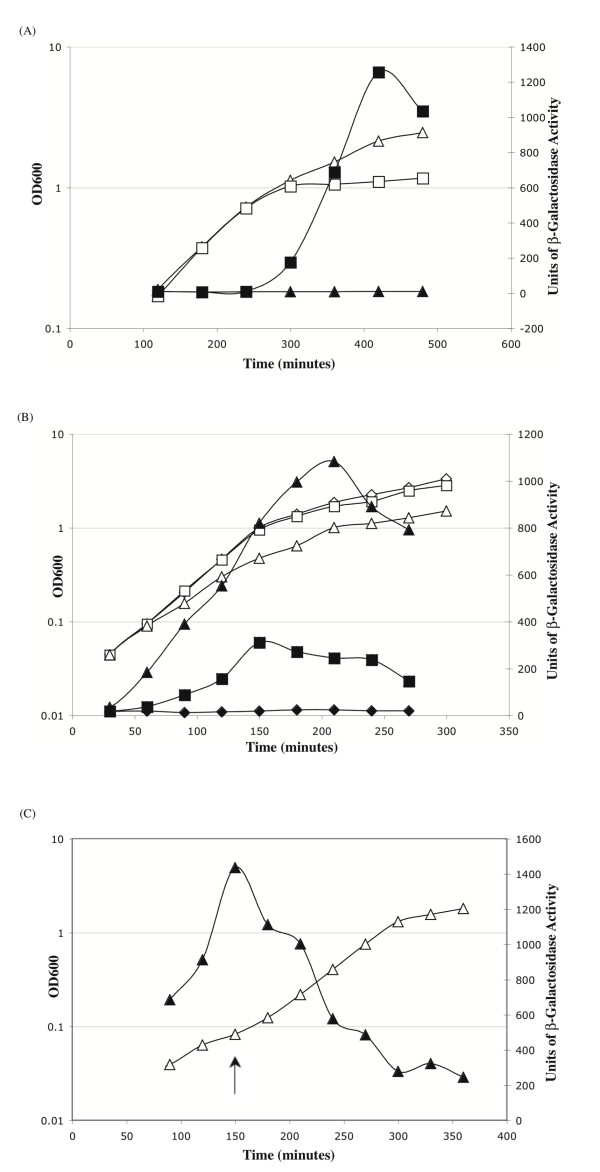
**Response of the *B. cereus lysK *T-box regulatory element to reduced tRNA^Lys ^charging**. Growth is represented by open symbols and β-galactosidase activity by closed symbols. Representative expression profiles are presented. Growth is represented by open symbols and β-galactosidase activity by closed symbols. (A) Growth and β-galactosidase accumulation in strain NF33 (P_*lysK*(T box) _*lacZ*) in minimal media. Strain NF33 was grown in minimal medium containing 100 μg/ml lysine (triangles) and 20 μg/ml lysine (squares). (B) Growth and β-galactosidase accumulation in strain BCJ367 (P_*lysK Tbox*_*lacZ *P_spac _*lysS *pMap65) in LB containing varying IPTG concentrations: 1 mM IPTG (diamonds); 250 μM IPTG (squares) and 100 μM IPTG (triangles). (C) Growth and β-galactosidase activity of strain BCJ367 in LB containing 100 μM IPTG. The IPTG concentration was increased to 1 mM at 150 minutes, indicated by the arrow.

The T-box element in the *B. cereus *and *B. thuringiensis *strains has a canonical structure [8], is highly conserved and controls expression of a class I LysRS (encoded by the *lysK *gene) of *Pyrococcal *origin [20]. Interestingly, the *lysK *gene is expressed predominantly during stationary phase in *B. cereus *strain 14579, whereas the class II LysRS is expressed during exponential growth of this bacterium [8]. To ascertain whether this T-box element is functional, expression of a P_*lysK*(T box) _*lacZ *transcriptional fusion (present in single copy at the *amyE *locus of the *B. subtilis *chromosome) was established under conditions of lysine starvation (strain NF33 is a lysine auxotroph) and LysRS2 depletion (strain BCJ367 has the endogenous *lysS *gene under the control of the IPTG-inducible P_spac _promoter). The results are shown in Figure 1. When strain NF33 is grown in lysine replete medium, only a low level of P_*lysK*(T box) _*lacZ *expression (~10 units of β-galactosidase activity) is observed (Figure 1A, squares). However growth in a lysine depleted medium (growth cessation occurs at ~ OD_600 _1 due to lysine deficiency) results in a high level of P_*lysK*(T box) _*lacZ *expression, with accumulation of ~1200 units of β-galactosidase activity. Importantly P_*lysK*(T box) _*lacZ *induction is coincident with the point of growth cessation due to lysine deficiency (Figure 1A). To confirm that increased P_*lysK*(T box) _*lacZ *expression is associated with increased levels of uncharged tRNA^Lys^, strain BCJ367 (P_spac _*lysS *P_*lysK*(T box) _*lacZ*) was grown in the presence of 1 mM IPTG, 250 μM IPTG and 100 μM IPTG. Growth of the cultures containing 1 mM and 250 μM IPTG was similar to that of wild-type strain 168 while growth of the cultures with 100 μM IPTG was reduced, presumably due to a decreased level of charged lysyl-tRNA^Lys ^(Figure 1B). Expression of P_*lysK*(T box) _*lacZ *is low (~10 units β-galactosidase activity) in cultures containing 1 mM IPTG. P_*lysK*(T box) _*lacZ *expression is initially low in cultures containing 250 μM IPTG but gradually increases with accumulation of ~200 units of β-galactosidase activity at the onset of stationary phase. However in cultures with 100 μM IPTG, P_*lysK*(T box) _*lacZ *expression increases throughout exponential growth with accumulation of more than 800 units of β-galactosidase during this period (Figure 1B). To confirm that this increased P_*lysK*(T box) _*lacZ *expression is due to increased levels of uncharged tRNA^Lys ^caused by LysRS2 depletion, a culture was grown for 150 minutes in the presence of 100 μM IPTG at which point, the IPTG concentration was increased to 1 mM (Figure 1C). Results showed accumulation of ~1200 units of β-galactosidase activity at 150 minutes but this level decreased subsequent to IPTG addition and continued to decrease for the remaining period of exponential growth (Figure 1C). It is noteworthy that the growth rate also increased upon IPTG addition (Figure 1C). As a control we established that P_*lysK*(T box) _*lacZ *expression is not induced by cellular depletion of phenylalanine showing that its induction shows the expected specificity (data not shown). These data show that the T box regulatory element found in the control region of the class I *lysK *gene of *B. cereus *strain 14579 is functional and responds to increased levels of uncharged tRNA^Lys ^in a canonical manner.

### A *B. subtilis *strain expressing the *B. cereus *class I LysK under T box regulatory control is viable

The rarity of the T box control of LysRS expression, and where found, occurs only in conjunction with a second cellular LysRS, prompted us to ask whether T box control of LysRS expression is compatible with viability. To address this question, *B. subtilis *strain NF54 (*amyE::*P_*lysK*(T box) _*lysK ∂lysS*) was constructed in which expression of the *B. cereus lysK *gene is under the control of its natural promoter and T box regulatory element in single copy at the *amyE *locus and the endogenous *lysS *gene is partially deleted (373 amino acids of LysRS deleted leaving only the C-terminal 126 amino acids) by a double cross-over event. It is important to note that in strain NF54 the P_*lysK*(T box) _*lysK *cassette is flanked by transcriptional terminators, ensuring that *lysK *expression is solely dependent on the P_*lysK*(T box) _promoter. This strain was successfully constructed and verified by PCR and Southern blot analysis and by sequencing of selected regions (data not shown). This confirms that in *B. subtilis *T box mediated control of LysRS1 expression is compatible with viability. To establish whether NF54 has a phenotype, growth profiles were established in rich (LB) and minimal media and compared with wild-type *B. subtilis *strain 168. Results show an increase in the generation time of strain NF54 during growth in LB medium: NF54 has a doubling time of ~31 minutes while that of wild-type strain 168 is ~22 minutes under these conditions. However strain NF54 does not grow in minimal medium whereas wild-type strain 168 has a generation time of ~76 minutes in this medium. To establish whether this growth phenotype was due to reduced tRNA^Lys ^charging, the P_*lysK*(T box) _*lacZ *was introduced into strain NF54 generating strain NF206. Reduced charging of tRNA^Lys ^in strain NF206 will result in increased β-galactosidase accumulation when compared with strain BCJ363 that has the P_*lysK*(T box) _*lacZ *contruct in an otherwise wild-type background (*ie*. with the endogenous class II *lysS*). Results show that 250-300 units of β-galactosidase accumulate during exponential growth of strain NF206, an ~20-fold increase over that observed in the control strain BCJ363. We conclude T box control of LysR1 expression is compatible with viability of *B. subtilis*. However such strains have a reduced growth rate in rich medium and cannot be propagated in minimal medium probably due to reduced tRNA^Lys ^charging.

### A *B. subtilis *strain with expression of the endogenous class II *lysS *under the control of the T box regulatory element is viable and indistinguishable from wild-type in terms of growth and tRNA^Lys ^charging

While T box control of LysRS1 expression supports growth of *B. subtilis*, the level of charged tRNA^Lys ^is reduced and there is a growth phenotype. However it is unclear whether this phenotype is caused by T box regulation of LysRS expression or is due to the *B. cereus *derived class I LysRS1 enzyme that is reported to be less efficient catalytically than its class II counterpart [21]. To distinguish between these possibilities and to further address the issue of T box regulation of LysRS, we constructed strain NF113 (*lysS*::P_*lysK*(T box) _*lysS*) that placed expression of the endogenous *B. subtilis lysS *gene under the control of the *lysK *promoter and T box element from *B. cereus *strain 14579. It is important to note that in strain NF113 the P_*lysK*(T box) _*lysS *cassette is flanked by transcriptional terminators ensuring that *lysS *expression is solely dependent on the P_*lysK*(T box) _promoter. Strain NF113 was successfully constructed and the relevant chromosomal regions verified by PCR and Southern blotting (data not shown) confirming that T box regulation of LysRS2 expression supports growth of *B. subtilis*. Importantly growth of strain NF113 in rich (LB) and minimal media (Spizizen salts) was indistinguishable from wild-type strain 168 (data not shown). The level of charged tRNA^Lys ^was assessed in strain NF113 by introducing the P_*lysK*(T box) _*lacZ *transcriptional fusion to generate strain NF205. Approximately 10 units of β-galactosidase accumulated during exponential growth of strain NF205 similar to control strain BCJ363 (data not shown). We conclude that T box control of *lysS *expression is compatible with growth of *B. subtilis*. It is likely that the growth and tRNA^Lys ^charging deficiency of strains NF54 and NF206 (containing T box regulated LysRS1) is caused by decreased efficiency of tRNA^Lys ^charging by LysRS1 rather than by T box control of its expression.

### The T box element associated with the *B. cereus *class I LysRS1 can be partially induced by asparagine starvation

**Figure 2:**
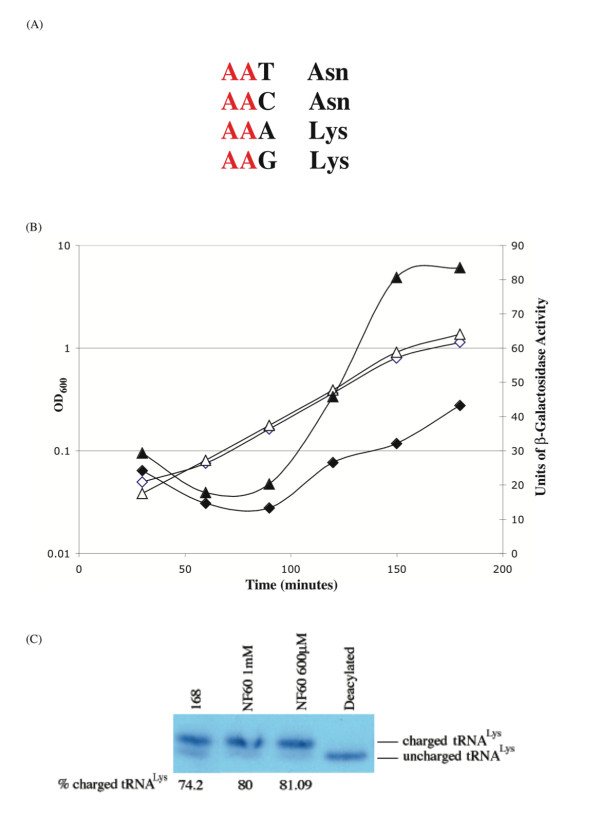
**Response of the *B. cereus lysK *T-box regulatory element to reduced levels of charged tRNA^Asn^**. A) The mixed codon box for lysine and asparagine. (B) Growth (open symbols) and β-galactosidase activity (closed symbols) of NF60 (P_spac _*asnS *P_*lysK Tbox*_*lacZ *pMAP65) in LB containing 1 mM (diamonds) and 600 μM (triangles) IPTG. (C) Northern analysis of tRNA^Lys ^charging in wild-type *B. subtilis *strain 168 and strain NF60 growing in LB media with the indicated IPTG concentrations. The percentage of charged tRNA^Lys ^is indicated beneath each lane. The profiles presented are representative.

The results presented show that while T box regulation of LysRS expression occurs very rarely and invariably in conjunction with a non-T box regulated paralogue, control of expression of the main LysRS by a T box mechanism is compatible with viability. This prompted us to question why T box regulation of LysRS expression does not occur more frequently. We noted that expression of neither LysRS nor AsnRS is regulated by a T box mechanism in *Bacilli *and that these two amino acids are encoded in a mixed codon box (Figure 2A). We therefore hypothesized that the T box element that controls expression of the class I LysRS1 of *B. cereus *may be inducible both by uncharged tRNA^Lys ^and tRNA^Asn^. A prediction of this hypothesis is that cellular depletion of charged tRNA^Asn ^may induce expression of P_*lysK*(T box) _*lacZ. *To test this hypothesis, strain NF60 (P_spac _*asnS *P_*lysK*(T box) _*lacZ*) was constructed containing the *asnS *gene under the control of the inducible P_spac _promoter (there is no *B. subtilis *asparagine auxotroph) and the P_*lysK*(T box) _*lacZ *to monitor induction. The growth profiles of NF60 cultures containing 1 mM and 250 μM IPTG were identical, but β-glactosidase accumulation differed significantly under these two conditions. Approximately 30 units of β-galactosidase accumulated during exponential growth of the culture containing 1 mM IPTG while more than 350 units of β-galactosidase accumulated during exponential growth of the culture containing 250 μM IPTG (data not shown). To exclude the possibility that depleting cellular levels of AsnRS leads to a concomitant increase in the uncharged tRNA^Lys ^level (and hence increased P_*lysK*(T box) _*lacZ *expression) we established the highest IPTG concentration at which some induction of P_*lysK*(*T *box) _*lacZ *occurred but at which growth of the culture was unaffected. The growth profiles of NF60 cultures containing 1 mM IPTG and 600 μM IPTG are identical (Figure 2B). However ~20-40 units of β-galactosidase accumulate during exponential growth of the culture containing 1 mM IPTG while more than 80 units of β-galactosidase accumulate during exponential growth of the culture containing 600 μM IPTG. Importantly the kinetics of P_*lysK*(T box) _*lacZ *expression differed in the two cultures: an increase in β-galactosidase accumulation is evident in the 600 μM culture that is not seen in the 1 mM IPTG culture. To verify that this induction is not due to an increased level of uncharged tRNA^Lys^, the cellular level of lysyl-tRNA^Lys ^was measured in wild-type strain 168 and in cultures of NF60 grown in 1 mM and 600 μM IPTG (Figure 2C). It is clear that the level of lysyl-tRNA^Lys ^is approximately the same (~80%) in both IPTG-containing cultures of NF60, a level that is higher than that observed (~74%) in cultures of wild-type *B. subtilis *strain 168 grown in the same medium (without IPTG). As an additional control, we measured P_*lysK*(T box) _*lacZ *expression and charged tRNA^Lys ^levels in cultures of strain BCJ367 (P_spac _*lysS *P_*lysK*(T box) _*lacZ) *growing in 1 mM and 600 μM IPTG. Approximately 20-30 units of β-galactosidase accumulated in both cultures and importantly the level of charged tRNA^Lys ^in both cultures was ~83% (data not shown).

We then sought to establish (i) if depletion of the cellular level of a charged tRNA leads to a general reduction in level of other charged tRNAs and (ii) if some level of cross-induction exists among T box elements controlling expression of AARS that charge the constituent tRNAs of mixed codon boxes in *B. subtilis*. To address both issues, transcriptional fusions of the promoter and T box element of the *pheS*, *ileS *and *trpS *AARS genes of *B. subtilis *with the *lacZ *reporter gene were constructed. Each fusion was introduced into strains auxotrophic for their cognate amino acids and into strains auxotrophic for the non-cognate amino acid in the mixed codon box. In each case, depletion for the cognate amino acid resulted in immediate induction of β-galactosidase expression while depletion for the non-cognate amino acid did not induce β-galactosidase expression to a significant level in any case (data not shown). These data show that depletion for an individual amino acid does not lead to a general increase in the level of uncharged tRNAs of other amino acids and that promiscuous cross-induction of T box controlled promoters by depletion of the non-cognate amino acid of a mixed codon box does not occur in *B. subtilis*.

We conclude that the T box element controlling expression of *lysK *encoding the class I LysRS1 of *B. cereus *strain 14579 displays some promiscuity of induction, being capable of responding to an increased level of uncharged tRNA^Asn ^in addition to uncharged tRNA^Lys^. However such promiscuous cross-induction is not a general feature of T box elements in *B. subtilis*.

## Discussion

### T box regulation of LysRS expression occurs rarely and in exceptional circumstances

The T box mechanism is widely employed to regulate AARS expression in a manner that is responsive to the level of uncharged cognate tRNA in the cell. Of 976 T box elements associated with regulation of AARS expression in 891 completely sequenced bacterial genomes identified in our analysis, potential T box control of LysRS expression was identified in only 4 bacterial species: T box elements were identified in all sequenced strains of *B. cereus *(except AH820) and *B. thuringiensis*, in association with a class I LysRS1 of *Pyrococcal *origin [8]; a T box element was identified in *C. beijerinckii *associated with a class II LysRS2 [17] and a T box element was identified in *S. thermophilum*, associated with a class I LysRS1 [16]. The T box elements in the *Bacillus *and *Clostridium *species are homologous: the T box elements of the *Bacillus *strains are ~92% identical while ~50% identity exists between the T box elements of the *Bacillus *and *Clostridium *species (see Additional file 1, Figure S1). However the T box element of *S. thermophilum *appears unrelated to the other T box elements (see Additional file 1, Figure S3). This is especially interesting since despite its high G+C (68.7%) content, *S. thermophilum *proteins are more similar to those of the low G+C Firmicutes such as *Bacilli *and *Clostridia *than to the high G+C Actinobacteria. In view of this, it is also interesting that among the homologous T box elements, those in the *Bacilli *are associated with a class I LysRS while the T box element in *C. beijerinckii *is associated with a class II LysRS. Thus T box regulation of LysRS expression appears to have evolved on two separate occasions, and one T box element has been conjoined with two different LysRS-encoding genes.

There are several interesting features about this cohort of T box regulated LysRS: (i) all bacterial species with a T box regulated LysRS have a second LysRS that is not T box regulated; (ii) the four T box elements in the phylogenetically related *B. cereus *and *B. thuringiensis *species are associated with a class I LysRS1 and display ~92% identity; (iii) the class I LysRS1 of *B. cereus *and *B. thuringiensis *is most closely related to LysRS1 from *Pyrococcal *species suggesting that a common ancestor of *B. cereus/thuringiensis *acquired it by a lateral gene transfer event [20]; (iv) the T box regulated LysRS1 in *B. cereus *strain 14579 is expressed predominantly in stationary phase [8] and (v) T box elements do not occur in *Archaebacteria*. The likely *Pyrococcal *origin of *B. cereus *LysRS1 and the absence of T box elements in *Archaebacteria *presents an interesting question as to how the regulatory sequence and structural gene were conjoined in this case. Perhaps tRNA^Lys^-responsive T box elements were more common in the ancestor of *Firmicutes *(supported by a similar T box element being associated with a class II LysRS2 in *C. beijerinckii*) and were selectively lost as controlling elements of the principal cellular LysRS, but were retained for control of ancillary LysRS enzyme expression. A second interesting possibility, especially in view of the fact that it can be induced by tRNA^Asn^, is that the T box element associated with *lysK *of *B. cereus *may have evolved from an element with a specificity determinant similar in sequence to that of lysine. These observations suggest that T box regulation may be unsuited for controlling expression of the housekeeping LysRS in bacteria and perhaps is only tolerated in additional copies of LysRS that play an ancillary role such as adaptation to stationary phase conditions as observed in *B. cereus*. Determining whether the other T box regulated *lysS *genes play an ancillary role requires further investigation. Notably, T box regulation of housekeeping aminoacyl tRNA synthetases is widespread, suggesting that it is some aspect of lysine metabolism that makes T box control of LysRS expression unsuitable as a regulatory mechanism.

### The LysRS1 T box element from *B. cereus *is functional and *B. subtilis *strains with T box control of LysRS1 and LysR2 expression are viable

The unknown provenance and functionality of the T box element, despite the reported theoretical capability to form canonical T box element structures [8] prompted us to verify that it was functional and to ask whether strains of *B. subtilis *expressing a single copy of LysRS1 or LysRS2 controlled by this T box element are viable. We chose to conduct this study in *B. subtilis *because of the paucity of relevant auxotrophic *B. cereus *strains and other difficulties with antibiotic resistance and transformability. However we consider *B. subtilis *to be a valid model system in which to conduct this study. Our results show that the T box element is functional and can be induced up to 120-fold in response to lysine- or LysRS-depletion but not by depletion of non-cognate amino acids. Also strains of *B. subtilis *with expression of the endogenous LysRS2 controlled by this T box element are viable, and could not be distinguished from *B. subtilis *wild-type strain 168 during growth in rich or minimal medium. While a strain of *B. subtilis *expressing LysRS1 controlled by the T box element from *B. cereus *strain 14579 is also viable, it displays a growth defect when grown in rich medium and cannot be propagated in minimal medium. However it is likely that these phenotypes result from the reduced catalytic activity of class I LysRS enzyme rather than from control of expression by the T box element. These results show there is no *a priori *reason precluding control of LysRS expression by a tRNA^Lys^-responsive T box element. It emphasizes the puzzling rarity of T box regulated LysRS expression and the restriction of its occurrence in *B. cereus *strain 14579 to controlling expression of a LysRS1 enzyme that plays an ancillary role in adapting cells to adverse conditions.

### The T box element controlling expression of LysRS1 in *B. cereus strain *14579 can be induced by an increased level of uncharged tRNA^Asn^

The unusual occurrence of tRNA^Lys^-responsive T box elements and the experimentally demonstrated viability of *B. subtilis *strains with T box regulated LysRS expression prompted us to investigate why T box regulation of LysRS expression is rare. We noted a tendency in *B. subtilis *for non-T box regulated AARS (ArgRS, AsnRS, GltRS, LysRS, MetRS, and ProRS) to charge tRNAs with amino acids encoded in mixed codon boxes (ProRS being an exception, not being encoded by a mixed codon box). This observation, together with its possible origin being a T box element that is responsive to a different tRNA, prompted us to investigate whether the T box element controlling LysRS1 expression in *B. cereus *might also be induced by depletion of asparaginyl-tRNA^Asn^. Our results show that cellular depletion of AsnRS in *B. subtilis *results in induction of the P_*lysK*(T box) _*lacZ*. We show that this induction is not caused by concomitant depletion of lysyl-tRNA^Lys ^since induction occurs when cellular levels of charged tRNA^Lys ^are high (Figure 2). Importantly, there is no direct link in the biosynthetic pathways of lysine and asparagine. Also, expression of P_*lysK*(T box) _*lacZ *does not occur when cells are depleted for phenylalanine, showing that induction displays the expected specificity for lysine starvation. These data show that the T box element controlling expression of LysRS1 of *B. cereus *can be induced by an increased level of uncharged tRNA^Lys ^and tRNA^Asn^. However such promiscuity of induction is restricted to this lysK-associated T box element since T box element control of expression of AARSs within mixed codon boxes is frequently found [17] and induction of the T box-controlled *pheS*, *ileS *and *trpS *genes was not observed in response to starvation for the non-cognate amino acid of the mixed codon box. The induction promiscuity of the *B. cereus *LysRS1-associated T box element might derive from its having evolved from a T box element that responded to a different tRNA. Such promiscuity may be tolerated since LysRS1 in *B. cereus *appears to have an ancillary role during stationary phase, or it may even be advantageous in that it makes LysRS1 expression responsive to a broader range of adverse nutritional conditions.

## Conclusions

The T box regulatory element makes expression of AARS responsive to the uncharged level of their cognate tRNA and is widely used among bacteria. However significant variability exists in the frequency with which expression of individual AARSs is controlled by this mechanism [15-17], this study. It is largely unknown why T box regulation of LysRS expression is found in only 4 bacterial species (*B. cereus*, *B. thuringiensis*, *S. thermophilum *and *C. beijerinckii*) while more than 140 instances of T box control of IleRS expression are documented. Moreover these four bacterial species with a T box regulated LysRS all have a second non-T box regulated LysRS. We report that two tRNA^Lys^-responsive T box elements exist: the first is found in the *Bacillus *and *Clostridium *species controlling expression of a class I LysRS1 in *Bacillus *but a class II LysRS2 in *Clostridium*; the second in *S. thermophilum *displays little homology to the first T box element and controls expression of a class II LysRS2. We established that the T box element associated with *lysK *expression in *B. cereus *strain 14579 is functional, but unusually responds to an increased level of uncharged tRNA^Lys ^and tRNA^Asn^. Since LysRS1 is expressed mainly in stationary phase, this unusual induction profile may make its expression responsive to a wider range of nutritional signals. We also demonstrated that *B. subtilis *cells, in which expression of the endogenous *lysS *is controlled by the *lysK *T box element from *B. cereus*, are viable and are indistinguishable from wild-type *B. subtilis *strain 168 in terms of growth and tRNA^Lys ^charging. Thus there appears to be no *a priori *reason why expression of the main cellular LysRS is not regulated by a T box element in *B. subtilis *(in fact expression of a majority of the AARS are T box regulated in *B. subtilis*), making the rarity of T box control of LysRS expression among bacteria even more puzzling.

## Methods

### Bacterial strains, media and growth conditions

Bacterial strains used in this study are listed in Table 2. Cells were grown routinely at 37°C in LB media, Spizizen's minimal media [22] or Basal Limitation media [23]. All cloning in *E. coli *was carried out in strains TG1 or TP611 [24,25]. Transformation of *B. subtilis *and *E. coli *was performed as described [26,27]. Antibiotics were used at the following concentrations: ampicilllin, 100 μg/ml; spectinomycin, 100 μg/ml; chloramphenicol, 3 μg/ml; erythromycin, 2 μg/ml; phleomycin, 1.5 μg/ml and kanamycin, 10 μg/ml. IPTG was added to cultures as indicated in the text.

**Table 2:** Bacterial strains and plasmids used in this work

Strains, plasmids	Relevant characteristics	Reference or source
Strains		
*E. coli*		
TG-1	*supE hsd*Δ5 *thi *Δ(*lac-proAB*) F'[*traD*36]	[24]
TP611	*recBC hsdR^-^M^- ^cyab10pcn*	[25]
		
*B. cereus 14579*	wild type isolate	[34]
		
*B. subtilis*		
168	*trpC2*	Laboratory stock
1A717	*amyE::erm *Em^R^	[32]
1A765	*trpC2 lys*	[35]
NF33	*trpC2 lys amyE*::pBCJ307 (P_*lysK *(T box) _*lacZ*) Cm^R^	This study
NF52	*trpC2 amyE*::pNF48 (P_*lysK *(T box) _*lysK*) Spec^R^	This study
NF54	*trpC2 amyE*::pNF48 (P_*lysK *(T box) _*lysK*) Spec^R ^D*lysS *Kan^R^	This study
NF58	*trpC2 amyE*::pBCJ307 (P_*lysK *(T box) _*lacZ*) Cm^R ^*asnS*::pNF40 (P_spac _*asnS*) Em^R^	This study
NF60	*trpC2 amyE*::pBCJ307 (P_*lysK *(T box) _*lacZ*) Cm^R ^*asnS*::pNF40 (P_spac _*asnS*) Em^R^pMap65 (*P_pe _-lacI*) Ph^R^	This study
NF113	*trpC2 lysS*::pNF112 (P_*lysK *(T box) _*lysS*) Cm^R^	This study
NF204	*trpC2 amyE::*pBCJ307 (P_*lysK *(T box) _*lacZ*) Em^R ^Cm^R^	This study
NF205	*trpC2 lysS*::pNF112 (P_*lysK *(T box) _*lysS*) Cm^R ^*amyE::*pBCJ307(P_*lysK *(T box) _*lacZ*) Em^R^	This study
NF206	*trpC2 amyE*::pNF48 (P_*lysK *(T box) _*lysK*) Spec^R ^D*lysS:*Kan^R ^*amyE*::pBCJ307(P_*lysK *(T box) _*lacZ*)Cm^R^	This study
BCJ363	*trpC2 amyE*::pBCJ307 (P_*lysK *(T box) _*lacZ*) Cm^R^	This study
BCJ366	*trpC2 amyE*::pBCJ307 (P_*lysK *(T box) _*lacZ*) Cm^R ^*lys*::pXZ2 (P_spac _*lysS*) Em^R ^	This study
BCJ367	*trpC2 amyE*::pBCJ307 (P_*lysK *(T box) _*lacZ*) Cm^R ^*lys*::pXZ2 (P_spac_-*lysS*) Em^R ^pMap65 Phl^R^	This study
		
Plasmids		
pBluescript2 KS(-)	cloning vector Ap^R^	Stratagene, La Jolla CA
pMutin4	integration vector for *B. subtilis*	[27]
pBCJ102	pBluescript based vector containing transcription terminator cassettes Ap^R^	[29]
pBCJ144	vector to replace part of *B. subtilis lysS *with Kan^R^	[29]
pBCJ307	vector with transcriptional fusion of *B*. *cereus lysK *promoter and T box with *lacZ *Cm^R^	This study
pDG268	vector to generate *lacZ *promoter fusions at the *amyE *locus by double crossover Ap^R ^Cm^R^	[26]
pDG1730	vector for integration at the *amyE *locus in *B. subtilis *Spec^R^	[30]
pXZ2	Vector to placing *B. subtilis lysS *under control of IPTG inducible Pspac promoter Em^R^	This study
pMUTIN-XZ	pMUTIN4 with the *lacZ *gene removed Em^R^	This study
pMap65	replicating *B. subtilis *plasmid encoding *penP-lacI *Phl^R ^Kan^R^	[28]
pNF30	plasmid with *B. cereus lysK *promoter and T box element in pBCJ102 Ap^R^	This study
pNF40	*B. subtilis asnS *promoter on 516 bp fragment in pMUTIN-XZ	This study
pNF48	*B. cereus lysK *promoter and *lysK *gene cloned into pDG1730	This study
pNF112	the *lysK *promoter and T box element (423 bp) fused to *B. subtilis lysS *(672 bp fragment)	This study

### General molecular biology methods

Standard DNA manipulations and cloning procedures were carried out as described [26]. Chromosomal DNA was isolated from *B. subtilis *and *B. cereus *using the chromosomal DNA purification kit from Edge Biosystems (Gaithersburg, MD) according to the manufacturerer's protocol. Plasmid DNA was isolated by a modified boiling lysis method [26] and further purified using the Concert Rapid PCR Purification System (Invitrogen, Carlsbad, CA), or the Genelute Plasmid miniprep kit (Sigma Aldrich, St Louis, MO, USA) according to the manufacturer's instructions. PCR amplification was performed using Taq polymerase (Invitrogen, Carlsbad, CA) or high fidelity KOD polymerase (Calbiochem-Novabiochem Corp. USA). Sequencing was carried out by MWG Biotech-Germany (Ebersburg, Germany) and GATC Biotech (Konstanz, Germany). Oligonucleotides used in this study (listed in Table 3) were purchased from MWG Biotech-Germany (Ebersburg, Germany) or Sigma-Aldrich (St. Louis, MO, USA). For Southern blot analysis DNA was transferred to Biodyne membranes (Pall Gelman, Ann Arbor, MI, USA) by vacuum blotting and crosslinked by UV exposure (150 mJ). Dig labeled probes (Roche, East Sussex, UK) were prepared as per manufacturer's protocol and hybridized to the filter using high concentration SDS buffers. Filter washes and probe detection were carried out using the Dig detection kit (Roche, East Sussex, UK).

**Table 3:** Oligonucleotides used in this study

Primer	Sequence (5'-3')
NF2F	**CGGAATTC**CAAATGGCTTCTCGATCC
NF2R	**CGGAATTC**GGTGAGTATTATCCTACTTTTGC
NF3R/2	CG**GGATCC**GCGTTGTGGTGAGTGATAAACGG
NF9R	CG**GAATTC**CTTGCGTTGCTTTTAACTGTGTT
NF15F	GGTGAGTAATATATGAGTCAAGAAGAGCATAACC
NF15R	TTGACTCATATATTACTCACCTCAATTATTTTTTG
NF16F	CCC**AAGCTT**CTTTCTTGTTTTGGAGGGAAATATG
NF16R	CCC**AAGCTT**CCTTCAGGTGCGCTTCCAGTC
NF36F	CG**GAATTC**CGATTTCGATTCGGAAAAGTTTG
NF36R	CG**GGATCC**GCTACTTCATACGCCCAATGC
T7	TAATACGACTCACTATAGGG
M13 rev	CAGGAAACAGCTATGACC

* Added restriction sites are shown in bold.

### Strain construction

To construct strain NF33, a 400 bp DNA fragment from the region upstream of *B. cereus lysK *was amplified by PCR using primers NF36F and NF36R, cut with *Eco*RI and *Bam*HI, and ligated into similarly restricted pDG268 [28] to produce the plasmid pBCJ307. pBCJ307 was inserted into the *amyE *locus of the *B. subtilis *lysine auxotroph strain 1A765 by double crossover to produce strain NF33.

In order to analyze the effect of a reduction of the cellular level of charged tRNA^Lys ^on expression of a P_*lysK*(T box) _*lacZ *fusion, strain BCJ367 was constructed. Plasmid pBCJ307 was integrated into the *B. subtilis *chromosome by a double crossover event at the *amyE *locus to produce strain BCJ363. To place the endogenous *lysS *gene of *B. subtilis *under IPTG inducible control, plasmid pMUTIN4 [29] was digested with *Sal*I and *Bsi*WI and eluted from an agarose gel to remove the 2 kb *lacZ *gene. The ends of the plasmid molecule were blunt ended using Klenow polymerase and religated, resulting in plasmid pMUTINXZ. A 670 bp DNA fragment encoding the end of the *yacF *gene was amplified with oligonucleotides NF2F and NF2R using *B. subtiliis *strain 168 chromosomal DNA as a template. This fragment was digested with *Eco*RI and inserted into the *Eco*RI site of pMUTINXZ, resulting in plasmid pXZ2. Plasmid pXZ2 was then integrated onto the chromosome of strain BCJ363 by a Campbell type event generating strain BCJ366 thereby placing expression of the *lysS *gene under the control of the IPTG inducible P_spac _promoter. To effect tight control of the P_spac _promoter, replicating plasmid pMap65 [30] that encodes a *lacI *gene, was transformed into BCJ366 to produce strain BCJ367.

Strain NF54 was made to assess whether a *B. subtilis *strain expressing a T-box regulated *lysK *gene was viable. A 1.95 kb fragment of the *B. cereus *chromosome encoding the *lysK *promoter, leader region and structural gene was generated by PCR using oligonucleotides NF36F and NF9R. This fragment was digested with *Eco*RI and cloned into the *Eco*RI site of plasmid pBCJ102 that has transcriptional terminators flanking the multiple cloning site, to generate plasmid pNF30 [31]. A 2567 bp fragment encoding the *lysK *promoter, T box element and structural gene flanked by transcriptional terminator sequences was amplified using the pBluescript T7 and M13 reverse primers and plasmid pBCJ102 as template. The ends of this fragment were phosphoryalted using T4 polynucleotide kinase (Promega) and it was then cloned into the *Eco*RV site of plasmid pDG1730 [32] to produce the plasmid pNF48. Plasmid pNF48 was integrated at the *amyE *locus of the *B. subtilis *chromosome by a double crossover event to produce strain NF52. The *lysS *gene of strain NF52 was then partially deleted by integration of plasmid pBCJ144 [31] into the chromosome by a double-crossover event, replacing part of *lysS *with a kanamycin resistance cassette, thereby generating strain NF54. In strain NF54, 373 amino acids of LysRS are deleted leaving only the C-terminal 126 amino acids). Importantly, in this strain the P_*lysK *(Tbox) _*lysK *construct is flanked by transcriptional terminators so that *lysK *expression is solely dependent on the P_*lysK *(Tbox) _promoter. To insert the P_*lysK *(Tbox) _*lacZ *reporter fusion into the chromosome of *B. subtilis *strain NF54, plasmid pBCJ307 was integrated at the *amyE *locus, thereby generating strain NF206.

To construct *B. subtilis *strain NF113, that has expression of the endogenous *lysS *gene under the control of the *lysK *promoter and T box element, a 423 bp DNA fragment encoding the *B. cereus lysK *promoter and T box element (generated using oligonucleotides NF36F and NF15R) was fused to a 672 bp fragment of the *lysS *gene (generated using oligonucleotides NF15F and NF3R/2) by overlapping PCR (using the outside primers NF36F and NF3R/2). This DNA fragment was then digested with *Eco*RI and *Bam*HI and cloned into *Eco*RI digested pBCJ102 [31] to generate the plasmid pNF112: the P_*lysK *(Tbox) _*lysS *insert is flanked by transcriptional terminators in this plasmid. Plasmid pNF112 was then integrated into the *B. subtilis *chromosome at the *lysS *locus by a Campbell-type event to produce the strain NF113. To introduce the P_*lysK *(Tbox) _*lacZ *reporter fusion into strain NF113, it was transformed with chromosomal DNA from strain NF204 that contains the P_*lysK *(Tbox) _*lacZ *reporter fusion at the *amyE *locus, thereby generating strain NF205. Strain NF204 was constructed by transformation of strain 1A717 [32] with pBCJ307. To construct *B. subtilis *strain NF60 in which expression of the endogenous *asnS *gene is placed under the control of the IPTG-dependent P_Spac _promoter and containing the P_*lysK*(T box) _*lacZ *fusion, a 516 bp DNA fragment encoding the *asnS *promoter region was amplified using oligonucleotides NF16F and NF16R, digested with *Hind*III and cloned into *Hind*III digested pMutinXZ to produce plasmid pNF40. Plasmid pNF40 was transformed into *B. subtilis *strain BCJ363 by a Campbell-type event to produce strain NF58. Plasmid pMAP65 (encoding the *lacI *gene) was then established in strain NF58 to ensure strict IPTG-dependent *asnS *expression, thereby generating strain NF60.

### Measurement of tRNA charging by Northern analysis

Establishing the level of charged tRNA^Lys ^was carried out as previously described [31]. *B. subtilis *tRNA^Lys ^was detected with an oligonucleotide probe complementary to nucleotides 26-51 that was labeled either with DIG oligonucleotide Tailing Kit (Roche, East Sussex, UK) or with biotin (New England Biolabs, USA). Detection used either the DIG labeling kit (Roche, East Sussex, UK) or the NEB blot phototope kit (New England Biolabs, USA) according to the manufacturer's instructions.

### Determination of β-galactosidase activity

Measurement of β-galactosidase activity was as previously described [33].

### Bioinformatic analysis

The genes encoding AARS proteins were identified in 891 bacterial genomes by homology with their orthologues of *B. subtilis *- for glutaminyl tRNA synthetases, the *E. coli *protein was used. Only proteins that displayed BLAST E-values of less than 10^-10 ^were retained for further analysis. The complete upstream region of each AARS-encoding gene was examined for the presence of the T-box motif TGGNACCGCG, allowing up to two mismatches in the last six positions. Sequences containing potential T-box sequences were then examined manually for their ability to form mutually exclusive terminator and anti-terminator DNA structures

## Authors' contributions

NF performed the experiments, analyzed the data and contributed to writing the paper, BCJ performed some experiments and contributed to writing the paper, GC performed the bioinformatic analysis and contributed to writing the paper and KD initiated the study, analyzed the data and contributed to writing the paper.

## Supplementary Material

Additional file 1 **Sequence alignment and putative structures of T box regulatory elements from *Bacillus cereus *(*lysK*), *Bacillus thuringiensis *(*lysK*), *Clostridium beijerinckii *(*lysS2*) and *Symbiobacterium thermophilum *(*lysS*)**. Figure S1 shows a sequence alignment of the T box regulatory elements associated with the *lysK *genes of *B. cereus *and *B. thuringiensis*. Figure S2 shows a sequence alignment of the T box regulatory elements associated with the *lysK *gene from *B. cereus *and the *lysS2 *gene from *C. beijerinckii*. Figure S3 shows a sequence alignment of the T box regulatory elements associated with the *lysK *gene from *B. cereus *and the *lysS *gene from *S. thermophilum*. Figure S4 shows a sequence alignment of the T box regulatory elements associated with the *lysS *gene from *S. thermophilum *and the *lysS *gene from *C. beijerinckii*. Figure S5 shows a putative structure for the T box regulatory element associated with the *lysK *gene from *B. cereus*. Figure S6 shows a putative structure of the T box regulatory element associated with the *lysS2 *gene from *C. beijerinckii*. Figure S7 shows a putative structure for the T box regulatory element associated with the *lysS *gene from *S. thermophilum.* Click here for file
