# Supplementary material for: The T box regulatory element controlling expression of the class I lysyl-tRNA synthetase of Bacillus cereus strain 14579 is functional and can be partially induced by reduced charging of asparaginyl-tRNAAsn
Source: BMC Microbiol. 2010 Jul 22;10:196. doi: 10.1186/1471-2180-10-196 (PMC2916919; doi:10.1186/1471-2180-10-196)
Supplement: Additional file 1 — Sequence alignment and putative structures of T box regulatory elements from Bacillus cereus (lysK), Bacillus thuringiensis (lysK), Clostridium beijerinckii (lysS2) and Symbiobacterium thermophilum (lysS). Figure S1 shows a sequence alignment of the T box regulatory elements associated with the lysK genes of B. cereus and B. thuringiensis. Figure S2 shows a sequence alignment of the T box regulatory elements associated with the lysK gene from B. cereus and the lysS2 gene from C. beijerinckii. Figure S3 shows a sequence alignment of the T box regulatory elements associated with the lysK gene from B. cereus and the lysS gene from S. thermophilum. Figure S4 shows a sequence alignment of the T box regulatory elements associated with the lysS gene from S. thermophilum and the lysS gene from C. beijerinckii. Figure S5 shows a putative structure for the T box regulatory element associated with the lysK gene from B. cereus. Figure S6 shows a putative structure of the T box regulatory element associated with the lysS2 gene from C. beijerinckii. Figure S7 shows a putative structure for the T box regulatory element associated with the lysS gene from S. thermophilum. [file 1471-2180-10-196-S1.PDF]

```
Bcer_lysK       AAACTAATATTTAA---GGCATAGAAGAGAAGAGTAGTTAAGAAAGAATACGTACAGAGA 57
BthurlysK       AAACTAATATGTAAAATGGCATGGAAGAGAAGAGTAGTTAAGAAAGAATACGCACAGAGA 60
                ********** ***   ***** ***************************** *******

Bcer_lysK       GCTCTGGTAGCTGAGAAAGAGCGGTATACATCTTAATGAAAAAAGACTTGGAGCTGCGCA 117
BthurlysK       GCTCTGGTAGCTGAGAAAGAGCGGTATACATCTTAATGAAAAAAGACTTGGAGCTGCGCA 120
                ************************************************************

Bcer_lysK       AGGAACTAATTTATTTAGGATGGTGCGACGGGTTCTCCCGTTATAGAGATAGGGTATAA 177
BthurlysK       AGGAACTAATTTATTTAGGATGATGCGACGGGTTCTCCCGTTATAGAGATAGGGTATAA 180
                ********************** *************************************

Bcer_lysK       GCATATTTTGCCGTACCTGAAGAGGTTAATATGGTGACATATTAACAAACTGAGGTGGTA 237
BthurlysK       GCGTATTTTACCGTACCTGAAGAGGTTAATATGGCGACATATTAACAAACTGAGGTGGTA 240
                ** ****** ************************ *************************

Bcer_lysK       CCGCGAAGCTAACAACTCTCGTCCTCAAGATGAATAATCTTGGGGGTGGGAGTTTTTTTG 297
BthurlysK       CCGCGAAGCTAACAACTCTCGTCCTCAAGATGAATAATCTTGGGGGTGGGAGTTTTTTTA 300
                ***********************************************************

Bcer_lysK       TTGCATAAAACTGGAAATGAGAAAC--AAAAAATAATTGAGGTGAGTAATAT 347
BthurlysK       TTGCATAAAATTGAAAATGTGAAAACAAAAAAACGATAGAGGTGAGTAACAT 352
                ********** ** ***** ****   ******  ** *********** **
```

Figure S1: Alignment of *lysK* T box containing leader sequences from *B. cereus* (Bcer_lysK) and *B. thuringiensis* (Bthur_lysK). The specifier codon and T box sequence are shown in red and purple respectively.

```
Bcer_lysK       AAACTAATATTTAAGGCATAGAAGAGAAGAGTAGTTAAGAAAGAATACGTACAGAGAGCT 60
Cbeij_lysS      ---CATATAGGTGGAAGATAATGGCTACAAGCTTTTG-GACAGGATACCCATTGAG---- 52
                   *  ***  *     ***   *  *  **   **  ** ** ****  *  ***

Bcer_lysK       CTGGTAGCTGAGAAAGAGCGGTATACATCTTAATGAAAAAAGACTTGGAGCTGCGCAAGG 120
Cbeij_lysS      --GGTA--TAAGTTAAACTAGAATTCAGATGG--GAATGAAAACCCAGCA-TGAATCAAG 105
                  ****  * **  * *   * ** **  *    ***  ** **   *   **    * *

Bcer_lysK       AACTAATTTATTTAGGGATGGTGCGACGGGTTCTCCCGTTATAGAGATAGGGTATAAGCA 180
Cbeij_lysS      TTTCACTTAATAGAGGTAGGATGTGACGGAATACCCCGTTATAGGTATAGAGTATCT-CG 164
                    * ** **  *** * * ** *****  *  **********  **** ****   *

Bcer_lysK       TATTTTGCCGTACCTGAAGAGGTTAATATGGTGACATATTAACAAACTGAGGTGGTACCG 240
Cbeij_lysS      TTTTTTGATGTACTTGAAGAGGTGTATACGGCAACGCATACATAAACTGAGGTGGTACCA 224
                * *****  **** *********  *** **  **  **  * ****************

Bcer_lysK       CGAAGCTAACAACTCTCGTCCTCAAGATGAATAATCTTGGGGGTGGGAGTTTTTTTG--T 298
Cbeij_lysS      CGAA-TTTATAACTTTCGTCCTCAAGATTAGAAATCTTGGAGGTGAAGGTTTTTTTTATT 283
                ****  * * **** ************* *  ******** ****   ********   *

Bcer_lysK       TGCATAAAACTGGAAAT-GAGAAACAAAAAATAATTGAGGTGAGTAATAT
Cbeij_lysS      TGTATTAAGGCAATATCAGAAGAATAACAAGTAAATCCGCTTTCTTGCCT
                ** ** **      *   **  ** ** ** *** *  * *   *    *
```

Figure S2: Alignment of T box containing leader sequences from *B. cereus* (Bcer_lysK) and *C. beijerinckii* (Cbeij_lysS). The LysRS in *B. cereus* is a class I enzyme while that in *C. beijerinckii* is a class II enzyme. The specifier codon and T box sequence are shown in red and purple respectively.

```
Bcer_lysK         -AAACTAATATTTAAGGCATA--GAAGAGAAGAGTAGTTAAGAAAGAATACGTACAGAGA 57
Sthermo_lysS      GAAGGGGGCGGGGGATGCCCTCCGCTGCGCAGGTTGTCCCGGGCACAGAGAGCGGAACGG 60
                   **           * **     *  * * **  *      *  * *    *   *  *

Bcer_lysK         GCTCTGGTAGCTGAGAAAGAGCGGTATACATCTTAATGAAAAAAGACTTGGAGCTGCGCA 117
Sthermo_lysS      ACGGTGCGAGTTCC----GACCCGCGGAGACCT--GCGCATAAGGCCCCGGAGCTGCCTC 114
                   *  **  ** *      ** * *   * * **    * * ** * *  ********

Bcer_lysK         AGGAACT---AATTTATTTAGGGATGGTGCGA---CGGGTTCTCCCGTTATAGAGATAGG 171
Sthermo_lysS      CCGAACCCCCGGCGCGCCGGGGCAGTAGACGAGGCCGGTTTCTCCCCCGATAC---CGGG 171
                    ****              ** *     ***   *** *******   ***      **

Bcer_lysK         GTATAAGCATATTTTGCC-----GTACCTGAAGAGGTTAATATGGTGACATATT----AA 222
Sthermo_lysS      AGCCAGGCGTTCTTCGTGCGGGAGCGCCGGGTGAGGTGGCCGCGCGGGTTCTCCCGCGG 231
                      * ** *  ** *       *  ** *  *****       * *   * *

Bcer_lysK         CAAACTGAGGTGGTACCGCGAAGCTAACAACTCTCGTCCTCAAGATGAATAATCTTGGGG 282
Sthermo_lysS      CCAAGTTGGGTGGTACCGCGGAGCAGA-GGCCTCCGTCCCGACGGCG---------GGAC 281
                  * ** *  ************ ***  *   *   *****  * *  *         **

Bcer_lysK         GTGGGAGTTTTTTGTTGCATAAAACTGGAAATGAGAAACAAAAAATA
Sthermo_lysS      GGAGGCCTTGTCATGTTTTCCGACTGTGGAGGAGGAGGATTGGCTGTA
                  *  **  ** *  ****     *   ****   *    *       **
```

Figure S3: Alignment of T box containing leader sequences from *B. cereus* (Bcer_lysK) and *S. thermophilum*. The LysRS in *B. cereus* is a class I enzyme while that in *S. thermophilum* is a class II enzyme. The specifier codon and T box sequence are shown in red and purple respectively.

```
Sthermo_lysS      -------GAAGGGGGCGGGGGATGCCCTCCGCTGCGCAGGTTGTCC--CGGGCACAGAGA 51
Cbeij_lysS        CATATAGGTGGAAGATAATGGCTACAAGCTTTTGGACAGGATACCCATTGAGGGTATAAG 60
                         *  *  *     ** * *   *   **  **** *  **   * *   * *

Sthermo_lysS      GCGGAACGGAC----GGTGCGAGTTCCGACCCGCGGAGACCTGCGCATAAGGCCCCGGAG 107
Cbeij_lysS        TTAAACTAGAATTCAGATGGGAATGAAAACCCAGCATGAATCAAGTTTCACTTAATAGAG 120
                      *   **     * ** ** *    ****     **     *  * *       ***

Sthermo_lysS      CTGCCTCCCGAACCCCCGGCGCGCCGGGGCAGTAGACGAGGCCGGTTTCTCCCCCGATAC 167
Cbeij_lysS        GTAGGATGTGACGGAATACCCCGTTATAGGTATAGAGTATCTCGTTTTTTGAT---GTAC 177
                   *       **        * **     *   ****  *   ** *** *       ***

Sthermo_lysS      CGGGAGCCAGGCGTTCTTCGTGCGGGAGCGCCGGGTGAGGTGGGCCGCGCGGGTT----- 222
Cbeij_lysS        TTGAAGAGGTGTATACGGCA-ACGCATACATAAACTGAGGTGGTAC-CACGAATTTATAA 235
                    * **    *  * *  *   **    *      ********  * * **  **

Sthermo_lysS      CTCCCGCGGCCAAGTTGGGTGGTACCGCGGAGCAGAGGCCTCCGTCCCG------ACGGC 276
Cbeij_lysS        CTTTCGTCCTCAAGATTAGAAATCTTG-GAGGTGAAGGTTTTTTTTATTTGTATTAAGGC 294
                  **  **    **** *  *   *   * *  *   ***  *   *          * ***
```

Figure S4: Alignment of T box containing leader sequences from *S. thermophilum* (Sthermo_lysS) and *C. beijerinckii* (Cbeij_lysS). The LysRS in both strains is a class II enzyme. The specifier codon and T box sequence are shown in red and purple respectively.

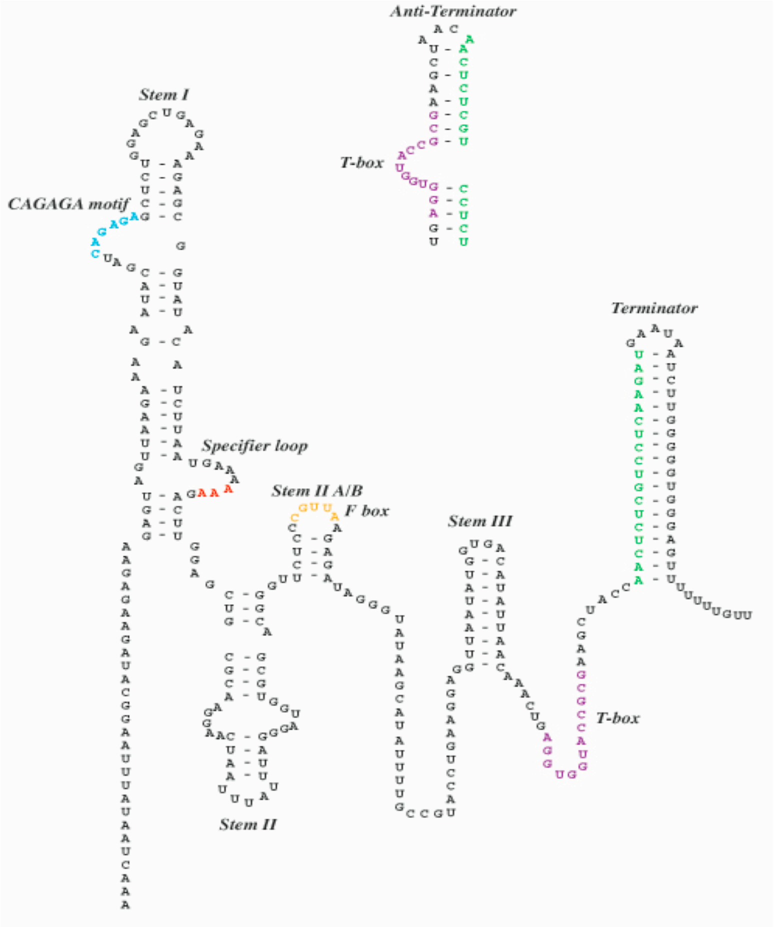

Figure S5: Putative structure of the leader sequence of *B. cereus lysK* showing conserved motifs in colour.

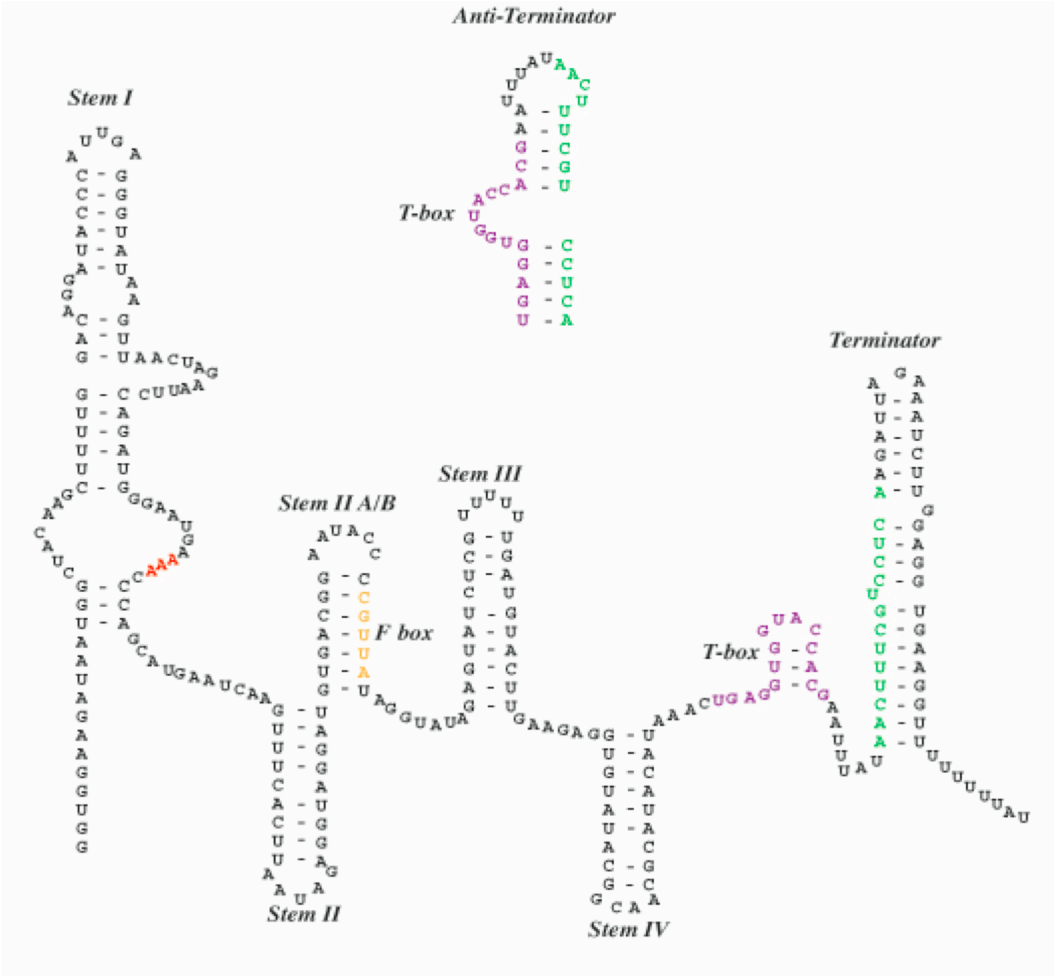

Figure S6: Putative structure of the leader sequence of *C. beijerinckii lysS2* showing conserved motifs in colour.

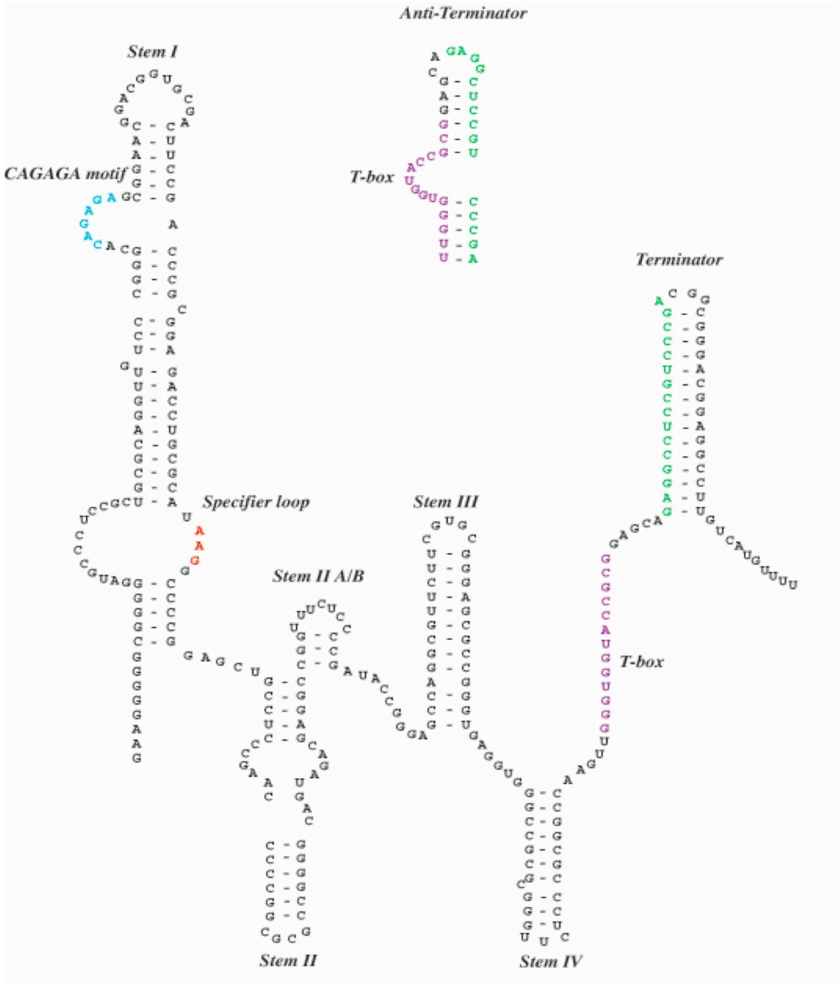

Figure S7: Putative structure of the leader sequence of *S. thermophilum lysS* showing conserved motifs in colour.
